# Stakeholder perspectives on a telemedicine referral and coordination model to expand medication treatment for opioid use disorder in rural primary care clinics

**DOI:** 10.1016/j.josat.2023.209194

**Published:** 2023-10-18

**Authors:** Allison J. Ober, Alex R. Dopp, Sarah E. Clingan, Megan E. Curtis, Chunqing Lin, Stacy Calhoun, Sherry Larkins, Megan Black, Maria Hanano, Katie P. Osterhage, Laura-Mae Baldwin, Andrew J. Saxon, Emily G. Hichborn, Lisa A. Marsch, Larissa J. Mooney, Yih-Ing Hser

**Affiliations:** a RAND Corporation, Santa Monica, CA, USA; b Department of Psychiatry and Biobehavioral Sciences, University of California, Los Angeles, CA, USA; c Semel Institute for Neuroscience and Human Behavior, Center for Community Health, University of California at Los Angeles, CA, USA; d Department of Family Medicine, University of Washington, Seattle, WA, USA; e Veterans Affairs Puget Sound Health Care System, USA; f Department of Psychiatry and Behavioral Sciences, University of Washington School of Medicine, Seattle, WA, USA; g Center for Technology and Behavioral Health, Geisel School of Medicine, Dartmouth College, Lebanon, NH, USA

**Keywords:** Opioid use disorder (OUD), Medication for opioid use disorder (MOUD), Telemedicine (TM), Primary care, Rural health, Implementation

## Abstract

**Introduction:**

Opioid overdose deaths are increasing rapidly in the United States. Medications for opioid use disorder (MOUD) are effective and can be delivered in primary care, but uptake has been limited in rural communities. Referral to and coordination with an external telemedicine (TM) vendor by rural primary care clinics for MOUD (TM-MOUD) may increase MOUD access for rural patients, but we know little about perspectives on this model among key stakeholders. As part of a TM-MOUD feasibility study, we explored TM-MOUD acceptability and feasibility among personnel and patients from seven rural primary care clinics and a TM-MOUD vendor.

**Methods:**

We conducted virtual interviews or focus groups with clinic administrators (*n* = 7 interviews), clinic primary care and behavioral health providers (8 groups, *n* = 30), other clinic staff (9 groups, *n* = 37), patients receiving MOUD (*n* = 16 interviews), TM-MOUD vendor staff (*n* = 4 interviews), and vendor-affiliated behavioral health and prescribing providers (*n* = 17 interviews). We asked about experiences with and acceptability of MOUD (primarily buprenorphine) and telemedicine (TM) and a TM-MOUD referral and coordination model. We conducted content analysis to identify themes and participants quantitatively rated acceptability of TM-MOUD elements on a 4-item scale.

**Results:**

Perceived benefits of vendor-based TM-MOUD included reduced logistical barriers, more privacy and less stigma, and access to services not available locally (e.g., counseling, pain management). Barriers included lack of internet or poor connectivity in patients’ homes, limited communication and trust between TM-MOUD and clinic providers, and questions about the value to the clinic of TM-MOUD referral to external vendor. Acceptability ratings for TM-MOUD were generally high; they were lowest among frontline staff.

**Conclusions:**

Rural primary care clinic personnel, TM-MOUD vendor personnel, and patients generally perceived referral from primary care to a TM-MOUD vendor to hold potential for increasing access to MOUD in rural communities. Increasing TM-MOUD uptake requires buy-in and understanding among staff of the TM-MOUD workflow, TM services offered, requirements for patients, advantages over clinic-based or TM services from clinic providers, and identification of appropriate patients. Poverty, along with patient hesitation to initiate treatment, creates substantial barriers to MOUD treatment generally; insufficient internet availability creates a substantial barrier to TM-MOUD.

## 1. Introduction

The opioid crisis continues to be of urgent national concern in the United States, with over 500,000 people dying from an overdose involving opioids between 1999 and 2020 (Centers for Disease Control and Prevention, 2022). Moreover, estimates suggest that overdose deaths from opioids increased by nearly 30 % during the COVID-19 pandemic, with an estimated 91,500 dying in 2020 alone (Centers for Disease Control and Prevention, 2022). A large body of evidence has demonstrated that first-line medications for opioid use disorder (MOUD) such as buprenorphine are effective (American Society of Addiction Medicine, 2020; Connery, 2015) and can be delivered in office-based settings such as primary care (Fudala et al., 2003; Gibson et al., 2003; Mintzer et al., 2007), but uptake has been limited in general and in rural communities in particular (Andrilla et al., 2019). Barriers to MOUD uptake in rural communities include few providers who deliver MOUD (Andrilla et al., 2019; Havens et al., 2018), lack of adjunctive psychosocial services for complex patients (Andrilla et al., 2017), stigma toward MOUD and people with opioid use disorder (OUD) (Richard et al., 2020), geographic barriers such as long distances to clinics (Green et al., 2021), and lack of buy-in for OUD treatment (Keyes et al., 2014). Wider implementation of MOUD is essential to address the growing disparities in OUD incidence and death rates being observed in several rural regions compared to urban areas (Rigg et al., 2018; Swann et al., 2021).

Provision of MOUD through telemedicine (TM-MOUD)—i.e., use of remote providers either from within or outside of a clinic via synchronous video teleconferencing—could help address some of these longstanding barriers. TM-MOUD also could provide a safety net during public health emergencies, as was the case during early phases of the COVID-19 pandemic. At the start of the pandemic, audio and video visits with physicians were critically important to managing the pandemic’s impact on primary care services (Uscher-Pines et al., 2021) including MOUD and other substance use treatment (Clarke et al., n.d.; Buchheit et al., 2021; Hunter et al., 2021; Langabeer et al., 2021; Uscher-Pines, Sousa, Raja, et al., 2020). Importantly, the pandemic precipitated relaxation of federal regulations, such as the requirement that MOUD be initiated through an in-person visit (“United States Code,” 2020), removing barriers to use of telemedicine (TM) to provide MOUD and promoting discussion about the importance of maintaining these changes to sustain improved MOUD access (Davis & Samuels, 2021; Huskamp et al., 2021). Unfortunately, rural clinics were less likely to adopt TM during the pandemic or sustain TM services once adopted, underscoring the persistence of disparities for rural communities (Cantor et al., 2021; Chu et al., 2021).

Despite anecdotal reports of successes and challenges in TM delivery of MOUD and other substance use treatment services during the pandemic (Clarke et al., n.d.; Busch et al., n.d.; Buchheit et al., 2021; Clark et al., 2021; Fiacco et al., 2021; Guille et al., 2021; Harris et al., 2020; Hughto et al., 2021; Hunter et al., 2021; Langabeer et al., 2021; Uscher-Pines, Sousa, Raja, et al., 2020), questions remain about how TM-MOUD can optimize treatment generally and in rural settings specifically. A few studies have found that TM-MOUD outcomes are similar to in-person treatment (Rubin, 2019; Zheng et al., 2017) or in some cases better (Vakkalanka et al., n.d.), and that TM-MOUD generally is considered acceptable by providers and individuals with OUD (Hunter et al., 2021; Uscher-Pines, Sousa, Raja, et al., 2020; Weintraub et al., 2018). Health centers have reported incorporating TM into various points along the MOUD continuum of care, including OUD identification, OUD diagnosis, MOUD initiation, and ongoing treatment and monitoring (Uscher-Pines, Raja, Mehrotra, & Huskamp, 2020). Allpayer claims data suggest that several TM models were in use prior to the pandemic for MOUD, ranging from exclusive use of TM (least used), to delivering non-medication components like counseling via TM (more common but not widely used), to using TM to conduct initial assessments and prescribe MOUD as a supplement to in-person services (most common) (Huskamp et al., 2018).

TM-MOUD generally may help expand access to MOUD, especially in rural communities where local capacity for in-person services often is limited. One promising option is TM-MOUD provision by vendors, i.e., private companies or health systems outside of clinics that offer MOUD and other services provided by primary care providers and behavioral health specialists. Models of TM that use vendors or other health systems outside of primary care clinics are considered standard practice for medical specialties that may not be available in rural or other low-resource settings, such as dermatology, endocrinology, psychiatry, and rheumatology (Uscher-Pines, Sousa, Palimaru, et al., 2020); however, TM has not been well-studied as a standard care for people with OUD. Partnering with an external TM-MOUD vendor or other provider external to the clinic could offer primary care clinics, particularly those in rural settings, much-needed additional capacity to offer treatment to their patients with OUD, but little is known about how referring patients to and coordinating with an external TM provider would fit into rural primary care settings.

**Fig. 1.**
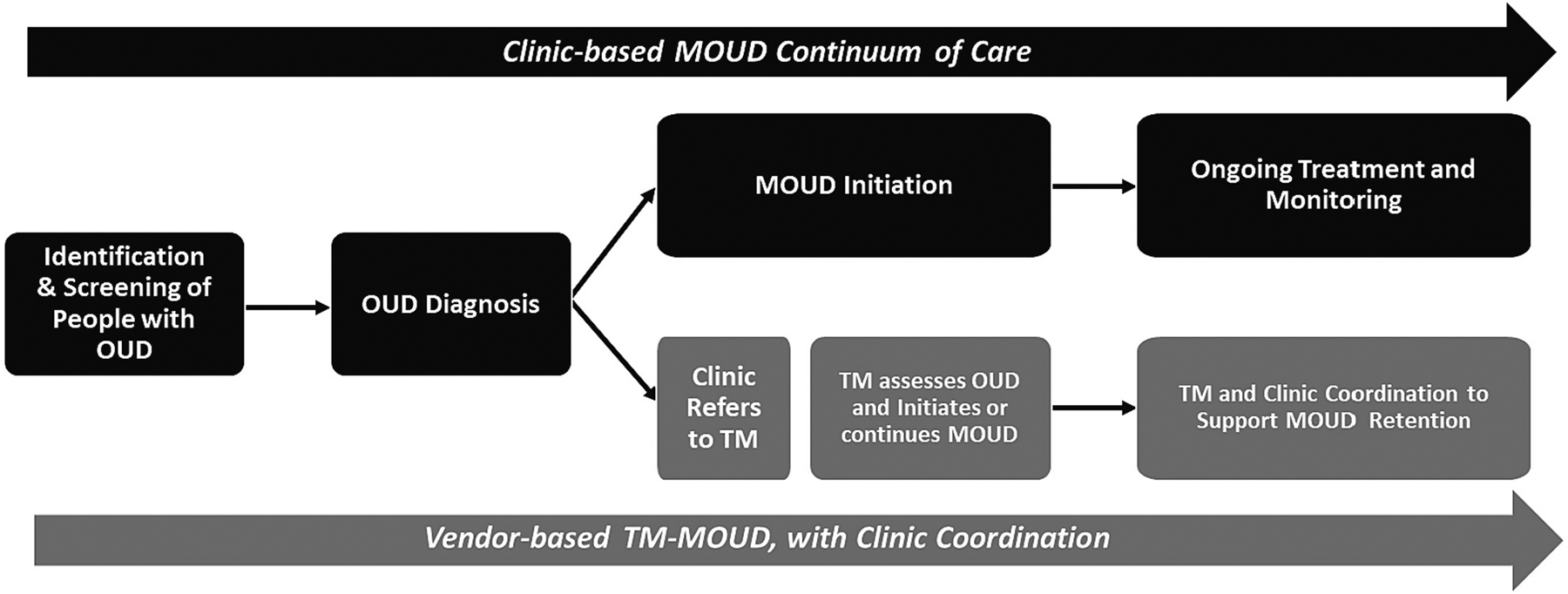
Clinic-based MOUD continuum of care and vendor-based TM-MOUD referral and coordination.

As part of a feasibility study developing and implementing a TM-MOUD referral and coordination model tailored to rural primary care settings (Hser et al., In Press), we used mixed methods (i.e., qualitative and quantitative) (Palinkas et al., 2011) to qualitatively explore stakeholder experiences of MOUD (primarily buprenorphine) care generally, and of the external TM-MOUD model. We also assessed quantitative ratings of the acceptability and feasibility of the model. The purpose of our qualitative work was to glean rich experiential data, while the quantitative ratings helped to broadly assess and quantify opinions using standardized measures. We explored experiences and perspectives of rural primary care clinic administrators, providers, and other clinic staff, and clinic patients with OUD, as well as administrative staff and providers working for the TM vendor involved in the feasibility study. Understanding multiple stakeholder perspectives can provide useful information about intervention-context fit, facilitate integration of new practices into healthcare settings, and improve patient outcomes (Boaz et al., 2018; Concannon et al., 2012). To guide our work and interpretation of findings, we followed a MOUD continuum of care framework that shows how referral to and coordination with a TM vendor can be integrated into the MOUD continuum of care in primary care settings (see Fig. 1) (Hser et al., 2021).

## 2. Methods

### 2.1. Setting and context

We conducted this project in a partnership between University of California – Los Angeles (UCLA), the RAND Corporation, and three Nodes of the National Drug Abuse Clinical Trials Network (CTN): the Greater Southern California Node, which led study protocol development, implementation, training, and evaluation, and the Pacific Northwest and Northeast Nodes, which assisted in recruiting the clinic sites and with local implementation. We conducted the feasibility study at seven primary care clinics in rural regions of the United States that are experiencing high rates of OUD (Rigg et al., 2018). The study recruited clinics in partnership with local CTN Nodes, including four from Washington/Idaho (Pacific Northwest Node) and three from Maine (Northeast Node). To be eligible for the overall feasibility study, clinics needed to offer MOUD services at their clinic, even if minimally, and to be able to identify at least 40 patients with an OUD diagnosis and a primary care visit during the study period. Clinics were required to designate a staff person (a “Care Coordinator,” typically a medical assistant or nurse) to oversee activities, with support from an identified provider (“Clinician Champion”), and to partner with the identified TM vendor to expand OUD and MOUD services. Clinics—typically medical assistants or other frontline staff—identified patients with OUD using a variety of methods (e.g., patient self-identification, electronic health record, screening). Clinic staff and providers worked with patients with OUD to determine whether they should receive MOUD at the clinic or by the TM vendor. TM vendor administrative and behavioral health staff assessed and initiated (or continued) MOUD for patients referred by the clinic. The TM vendor provided reports on patient progress to clinic staff and providers, and the TM vendor administrative and clinic staff coordinated to ensure patients initiated and maintained their connection with the TM vendor. To facilitate collaboration, the study developed a service delivery protocol in which clinic specified the staff and procedures for screening, diagnosing, and referring to TM.

The private TM vendor selected for participation in this study provided a variety of specialty services via remote videoconferencing, including MOUD and treatment for other substance use disorders, as well as mental health and pain management services. MOUD services included OUD assessment and diagnosis, prescription of MOUD (sent to local pharmacies to be filled for patients), follow-up and monitoring with patients, individual and group psychotherapy, and collection of urine drug tests (either through drug tests mailed to patients to be conducted on televideo, or referral to a local lab) to monitor patients’ use of medications and other substances. Patients used an internet-enabled device (e.g., computer, tablet, smartphone) to access the vendor’s services; patients who lacked such access at home could connect with vendor services from another location, including at their primary care clinic. One clinic provided smart phones to patients if they wanted to access TM-MOUD but didn’t have a phone.

Study participants included clinic personnel and patients from the seven feasibility study clinics who were invited to complete a research interview or focus group, as well as administrative staff and providers working with the private TM vendor. To participate, all participants needed to be 18 years or older and identified by their clinic or vendor as an administrator, provider, staff member, or patient.

### 2.2. Procedures

We used a QUAL + Quan study design (Palinkas et al., 2011), separately collecting qualitative and quantitative data and then integrating the findings. Our primary emphasis was on the qualitative data, which were more extensive. The quantitative data served a complementary function by quantifying perceptions of clinic-based- and TM-MOUD. The Biomedical Research Alliance of New York (BRANY) IRB approved all recruitment and data collection procedures (Protocol #19-PRS-435-709)—the single IRB for this multi-institutional project. Recruitment began after the first clinic had launched the feasibility study in July 2020 and continued through July 2021. Due to delays caused by the COVID-19 pandemic, some participants had not yet begun the feasibility study while others had experienced it for several months. To recruit clinic personnel and patients, research staff worked with Care Coordinators to distribute information about focus group and interview opportunities to potential participants. To recruit TM vendor personnel, research staff obtained a list of relevant personnel from the vendor, sent emails inviting them to participate, and coordinated scheduling. Once a focus group or interview was scheduled, research staff sent a Zoom videoconference meeting invitation that included an information sheet about the study. PhD-level researchers with expertise in substance use treatment, TM, implementation science, and qualitative methods contributed to the interview guide and facilitated focus groups and interviews. The facilitator began each focus group or interview by obtaining verbal consent from each participant to participate and be recorded, then proceeded through the focus group or interview guide. Each activity took approximately 1 hour to complete. For the focus groups, a second research staff person took notes; facilitators took their own notes for individual interviews. The study audio-recorded all focus groups and had them transcribed verbatim.

Following completion of the focus group or interview, we collected demographic information and a standardized rating scale that measured acceptability of clinic-based MOUD and TM-MOUD. The study sent clinic and vendor personnel a brief electronic survey; the facilitator asked the patients the questions at the end of their interview. Patients received a $50 electronic gift card as compensation and staff and providers received a $100 electronic gift card as compensation (if permitted by their organization’s honorarium policies), either upon completion of the survey or after 10 business days following their focus group or interview. Survey completion was not required to obtain the incentive.

### 2.3. Measures

Our use of mixed methods balanced in-depth exploration of experiences with MOUD and acceptability and feasibility of a vendor-based TM-MOUD model (qualitative measures) with collection of standardized information across all participants (quantitative measures), allowing for a rich, detailed understanding of stakeholders’ experience and perspectives.

#### 2.3.1. QUAL – focus groups and interviews

We developed qualitative, semi-structured guides for the focus groups and we designed interviews to elicit information about participants’ experiences with clinic-based MOUD generally and TM-MOUD during the feasibility study and about overall acceptability and feasibility of the vendor-based TM-MOUD referral and coordination model. We included questions about clinic-based MOUD to understand whether the MOUD continuum of care is generally acceptable and feasible. We used parallel questions for all clinic personnel (i.e., administrators, providers, staff) but tailored the questions to best fit participants’ respective roles (e.g., we only asked prescribers about diagnosing OUD and prescribing MOUD). Question topics included experiences providing clinic-based care for people with OUD (including experiences with screening for OUD to identify new patients with OUD, diagnosing OUD, and providing clinic-based MOUD) and thoughts about and experiences with the TM-MOUD vendor and the referral and coordination model. Interview questions for patients focused on their experiences receiving OUD services at the clinic and elsewhere (including universal screening and MOUD treatment), and their thoughts on (or experiences with, if relevant) working with a private TM vendor. Interviews with TM vendor staff and providers primarily focused on opinions about and experiences with the TM-MOUD referral and coordination model.

#### 2.3.2. Quan – survey questions

To complement our qualitative data, we collected standardized ratings from focus group and interview participants, focused on overall acceptability of MOUD and TM-MOUD. Clinic providers, staff, and administrators provided acceptability ratings of MOUD and of TM for MOUD. TM vendor staff and personnel rated the acceptability of the TM-MOUD primary care referral and coordination model. We adapted the survey items from three companion measures focused on the fit or match of an evidence-based practice (Weiner et al., 2017); the measures were four items each (i.e., 12 total), but we selected four items from across the measures to minimize respondent burden. We refer to our composite measure as “acceptability,” and included the following items “Use of [MOUD/TM-MOUD] meets my approval,” “I welcome use of [MOUD/TM-MOUD] at this clinic,” “[MOUD/TM-MOUD] is a good fit for this clinic,” and “It is/will be possible to offer [MOUD/TM-MOUD] at this clinic.” All questions use a five-point Likert scale (1 = completely disagree, 5 = completely agree).

For patients, we asked three questions to capture acceptability of MOUD and TM-MOUD (“Medication/Telemedicine is a good way to treat opioid problems”), as well as willingness to receive TM-MOUD (“I would consider continuing to get my medication for opioid use disorders using telemedicine”). We based these questions on the original Weiner et al. (2017) items.

### 2.4. Analysis

#### 2.4.1. Qualitative data analysis

We conducted rapid analysis of the qualitative data. Rapid analytic methods focus on identifying actionable insights rather than in-depth theory development or inferring meaning (Hamilton & Finley, 2019). Studies have shown that themes generated by rapid versus conventional, in-depth analysis to inform implementation are highly similar (Taylor et al., 2018). This approach enabled us to rapidly analyze our findings to inform ongoing implementation of the feasibility study.

Our procedures were as follows: First, following completion of each interview or focus group, the facilitator or note-taker recorded summary notes within a shared Excel spreadsheet. These notes captured key information shared by participants, organized within the major questions in the qualitative guides. Second, all facilitators individually reviewed their transcripts to ensure accuracy and completeness of the relevant summary notes. Third, the first and second authors used conventional content analysis (Hsieh & Shannon, 2005) to synthesize summary notes into general themes that captured the content of responses across participants. We identified themes based on their cohesiveness and prevalence across participant responses, but also incorporated inconsistent perspectives (i.e., negative case analysis) when relevant. We also identified more nuanced subthemes that focused on a specific aspect of or helped to explain a theme. To qualify as a theme, the concept had to appear in three or more transcripts. All facilitators met to review the initial list of themes and subthemes and discussed each until reaching consensus. Fourth, to confirm the themes and sub-themes, the first and second author reviewed all transcripts. During transcript review, we made iterative modifications to the themes and sub-themes so that they best reflected participants’ own words and selected exemplar quotes for each. We also organized themes within the MOUD care continuum domains in Fig. 1 (OUD Screening and Identification, OUD Diagnosing, Clinic-based MOUD, and Referral & Coordination), with an overarching theme for each care continuum domain. All facilitators had the opportunity to review the final groupings and provide additional feedback. Finally, we invited clinic personnel to participate in voluntary webinars to validate, or “member-check” themes identified by the researchers. While only five clinic personnel attended these webinars, participants who did attend concurred with the identified themes and subthemes regarding clinic personnel perspectives. Due to logistical barriers to follow-up, we were not able to member-check patient or vendor themes.

While our sample of 9 interviews and 15 focus groups with clinic personnel is beyond the sample size within which saturation is likely possible to achieve—the point at which qualitative data collection can stop because additional data do not result in meaningful changes in themes (Guest et al., 2006; Guest et al., 2016)—our samples for patients and vendor personnel were smaller. Nevertheless, we found that themes did overlap across stakeholder groups, suggesting that we captured key perspectives.

#### 2.4.2. Quantitative data analysis

Using Microsoft Excel and STATA 14 (StataCorp, 2013), we calculated descriptive statistics (e.g., *Ms, SDs,* frequencies, ranges) for survey items, including demographic information and rated perceptions of acceptability. For the clinic and vendor personnel surveys, we took average ratings on the acceptability items for each (MOUD, TM, or referral and coordination model). Internal consistency of the measure was high for all elements (α = 0.90–0.95). For patient surveys, we examined the item ratings separately. Our sample was too small to allow for adequately powered statistical tests for differences in ratings by element or participant type; instead, we created graphs of the mean ratings by element and participant type to visually inspect patterns in the data.

#### 2.4.3. Integrating QUAL + Quan findings

Within our QUAL + Quan design, we examined how patterns of quantitative ratings were consistent with (or extended upon) our qualitative themes, rather than interpreting the limited quantitative data in isolation.

## 3. Results

### 3.1. Participants

We conducted one interview and seven focus groups with clinic providers (i.e., primary care and behavioral health; *n* = 30 participants), one interview and eight focus groups with other staff (e.g., medical assistants, registered nurses; *n* = 37 participants) including two with the study’s Care Coordinators, and individual interviews with an administrative leader (e.g., CEO, CMO, program manager; *n* = 7 participants) from the seven clinics. We also completed 16 interviews with patients. From the TM vendor, we conducted four interviews with administrative staff, nine with prescribing providers, and eight with behavioral health providers. Of 111 total participants, 89 % completed the follow-up survey; minimal differences occurred in survey response rates across all participants (χ^2^ = 2.20, *p* = 0.33). Participant demographics for participants who completed the survey are shown in Table 1.

### 3.2. Stakeholder perspectives on the MOUD continuum of care and TM-MOUD referral and coordination model

We identified 11 themes from focus groups and interviews across clinic personnel, patients and vendor personnel. The text that follows discusses themes and exemplar quotes within categories of the MOUD continuum of care, and the TM-MOUD vendor referral and coordination model (Fig. 1).

#### 3.2.1. OUD screening, identification & diagnosis

*Theme 1: Screening for OUD has workflow implications; successful screening/identification of patients with OUD requires time and a variety of approaches*. Clinic administrators, providers, and staff believed screening patients for OUD is worthwhile, and necessary for identifying patients with OUD. However, many noted substantial workflow implications to implementing new, universal OUD screening practices. Examples of workflow implications included screening requiring extra time due to its sensitive nature and interfering with the clinic schedule, as described by this clinic provider:

"We had to cut [screening at every visit] back because it was so disruptive of our schedules. So we started doing it just on principal exams. So, basically, if someone’s come in for routine visits or something, they’re not getting screened, which is a problem. But if they could get them here 20 minutes early for their appointment, that would be okay."

(Clinic Provider, FG9)

All clinic stakeholder types suggested that successful OUD screening likely would improve with practice and increasing staff buy-in and that experimenting with a variety of approaches, including those that allow for more privacy, might help find the approach with the best fit for providers and patients. An example of one such approach was a more natural and targeted process for identifying patients with OUD than universal screening, as this provider explained:

"My personal experience has been times in which problems have come up more organically, like difficult problems have come up more organically in conversation or as a side note, ‘Hey, listen, you know, I saw you in the ER the other day, and, yeah, I noticed your opioid screening was positive on your urine drug screen’…those kind of settings where it’s been more organic have yielded better results with individuals than screening tools have."

(Clinic Provider, FG15)

*Theme 2: Patients may not support screening or disclose opioid use during screening*. Clinic stakeholders, including patients, noted that patients may not be willing to screen or to answer questions about opioid use truthfully. Reasons patients may not disclose their use included that patients may not be ready to start treatment, screening infringes on patients’ privacy, and/or patients worry about the consequences of disclosing their opioid use. This clinic provider described patients’ reactions to OUD screening:

"I would say at least twice that I have a patient ask why we’re screening, and what we’re tryin’ to do with that information? And why exactly we’re trying to pin everybody as a drug user. I mean, that was the words they used is, ‘Why are you tryin’ pin everybody as a drug user here’"

(Clinic Provider, FG15)

*Theme 3: OUD diagnosis coding in the electronic health record (EHR) is inconsistent and not always accurate*. Although no extensive discussion in interviews and focus groups occurred about the process of diagnosing patients with OUD, when the topic was discussed, agreement existed among providers that consistent use of OUD diagnosis codes in the EHR is problematic because of multiple OUD diagnosis codes in EHR and lack of consistent guidelines on which codes to use.

#### 3.2.2. Clinic-based MOUD

*Theme 4: Transportation issues, limited community resources and poverty impede access to MOUD and ancillary treatment*. All participants mentioned transportation as a substantial barrier to patients receiving MOUD in clinics, with many patients living a substantial distance from their clinics. The distance makes travel to the clinic a challenge, particularly during bad weather. As one patient described:

“If you have a drug problem and you’re in <City>, you have to drive down here four hours, get your medicine and drive home. That’s an eight-hour day. That’s a work job.”

(Patient, PI9)

Patients also described a lack of resources and their challenges trying to receive behavioral health services, even via TM:

“I’ve been referred for mental health now, but we’re havin’ a hard time out here in <City> gettin’ it together. I’ve been trying to get into somewhere to see, even through Zoom, to see somebody, or Skype or whatever it is. And we’ve been running into brick walls, so there’s—not a lot of care in terms of that.”

(Patient, PI17)

All participant types noted that poverty among patients also substantially impedes OUD treatment in the clinic, primarily due to lack of adequate transportation and money for gas, medication costs, and lack of insurance coverage.

*Theme 5: Patients are ambivalent about OUD treatment.* Participants also described how ambivalence about treatment for an OUD substantially impedes MOUD initiation; stigma and mistrust were identified as factors that may contribute to patient ambivalence. As expressed by this patient:

“I didn’t wanna do Suboxone only because I assumed that, once you’re on Suboxone, and every doctor that you go to, if you see that you’re on Suboxone, then they’re gonna assume you had a drug problem and nix you. So that was my biggest hurdle was I didn’t want to have Suboxone on my medical record.”

(Patient, PI14)

*Theme 6: There is lack of buy-in for MOUD and treating OUD patients among some clinic providers*. Participants indicated that lack of buy-in at the clinic level is a barrier to MOUD implementation in general. Some noted that provider and staff ambivalence about using medication to treat OUD still exists and could slow expansion of MOUD. As stated by this provider,

"I’m concerned with Suboxone, which is still a narcotic, correct? …Yeah. Well, methadone was gonna fix this before, and…to me, it just feels like we’re just jugglin’ narcotics. We’re not doin’ anything other than that."

(Clinic Provider, FG10)

Participants also noted that some providers are ambivalent about treating people with OUD more generally, for example due to concerns about workload and clinical competence, difficulties working with patients who have OUD and associated behavioral problems, or lack of training and understanding about OUD as a chronic disease (subthemes). As noted by this clinic administrator:

“I think for staff—the patients are challenging to work with. Even in the program, some days, patients will come in and just be emotionally upset and angry, and you know, they’re not getting what they want"

(Clinic Administrator, SI17).

#### 3.2.3. TM-MOUD vendor referral & coordination

The overarching theme among all participants regarding a TM-MOUD referral and coordination model through primary care clinics was that this model generally fits with current clinic and TM vendor practices and can be sustained. However, clinic and vendor personnel alike noted that successful implementation requires time, investment, clear communication, and trust from both primary care and vendor personnel and providers.

*Theme 7: Vendor-based TM-MOUD can reduce clinic capacity/overflow challenges and improve MOUD access*. Most clinic providers and staff (a mix of those who had already worked with the feasibility study and TM vendor, as well as those who had less exposure to the study) felt that a TM-MOUD referral and coordination model ultimately could reduce overflow burden at clinics with high prescribing and could expand patient access to MOUD at clinics with little to no prescribing capacity. As stated by this staff member who had experience with the model:

"It has given us an avenue to treat these patients that we weren’t treating before. There may have been some conversations with patients. But I mean, until we had this program, we didn’t even have opioid use disorder as something that we even charted. And so it’s definitely made us aware and given us an avenue to treat our patients."

(Clinic Staff, FG12)

*Theme 8: Vendor-based TM-MOUD could benefit some OUD patients, but not all; criteria for appropriate referrals are needed*. All stakeholders thought that vendor-based TM-MOUD through clinic referral and coordination could benefit some patients by offering more privacy than the clinic, scheduling flexibility to patients who may need services outside of regular clinic hours and expanded access to behavioral health such as individual or group therapy. This patient summed up the flexibility benefit this way:

“’Cause it eliminates the biggest problem, which is the getting to and from wherever you need to be. If you can do it from your own home—I mean, people are more likely to do something if they don’t have to go and put a lot of huge effort into going somewhere. You know?”

(Patient, PI9)

Another participant captured how TM-MOUD can alleviate the privacy problem some people with OUD experience in rural settings:

“[This is] a very small town, and I think that just that whole confidentiality thing. Like I said, when you walk through the door, everybody knows who you are … so I think [vendor] takes care of that issue as well”

(Clinic Administrator, SI13).

Benefits notwithstanding, several providers and patients felt strongly that TM-MOUD was best for patients who were already stable on MOUD. Definitions of “stable” varied, with some describing “stable” patients as those who had already gone through buprenorphine induction and were stabilized on the medication, while others focused on mental health stability or stability in living conditions. As one patient explained:

“In the later days of somebody’s recovery, I could see [TM-MOUD]. I don’t believe that initially, that would be a great way to start it.”

(Patient, PI7)

Relatedly, some clinic providers as well as patients were concerned that TM-MOUD might not provide sufficient oversight and accountability to patients who might not be able to adhere to TM-MOUD:

“Certain people would probably take advantage of the system and try to get away with things that are not allowed, you know, if they were to go in and do the [urinalysis] and stuff”

(Patient, PI8).

TM-MOUD providers expressed their view that limited patient readiness for MOUD vendor requirements (e.g., attendance in group and individual therapy sessions, attending classes to make up for missed sessions) can impede retention in TM-MOUD. Participants noted that although many of these issues may result in high no-show rates to TM appointments, they are similar to barriers to OUD treatment in brick-and-mortar treatment centers. (Of note, some respondents may not have been aware of low barrier models that do not impose such requirements (Jakubowski & Fox, 2020).) Several TM providers also suggested that patients who can succeed at receiving OUD services via TM are tech-savvy, have access to technology, and are willing to engage in MOUD treatment as well as TM-MOUD specifically.

Some participants, although more so clinic personnel than patients, were concerned about whether patients would support referral from their primary care clinic to receive treatment from an external TM-MOUD provider. Some clinic personnel thought patients might not trust an external TM-MOUD vendor or they might be resistant to TM more generally. As explained by one clinic provider:

“… Given a choice between an in-person meeting with consistency, people that they know in the community and develop a relationship with and, you know, a third party or a provider done through telemedicine…given that choice, I’m not sure where people would land on that."

TM-MOUD providers felt agreement should exist between clinic and TM-MOUD providers about appropriate patients for referral to TM-MOUD.

*Theme 9: Limited provider buy-in, and inadequate engagement and preparation, may result in TM implementation and sustainability challenges; pre-referral communication is needed*. Providers and staff at clinics who had significant involvement with the feasibility study reported implementation challenges related to limited engagement and preparation for the TM-MOUD referral and coordination model. Examples included low clinic provider trust in the services offered by TM providers, conflicting prescribing cultures and philosophies, fear of losing control and relationships with patients, and little understanding of the relative advantage of offering MOUD through a TM-MOUD vendor instead of through clinic providers. Low provider trust and conflicting prescribing practices were expressed by one provider this way: “*The prescribing practices I’ve had several concerns with, but at that point, I’m kinda stepping back and letting them do it on their own license.”* (Clinic Provider, FG9).

Another provider questioned the relative advantage of referring patients to an external TM-MOUD vendor over clinic-based MOUD (either in-person or via TM):

“I guess, I don’t have a great or a clear understanding of why we would be referring people out unless it was an overflow issue— I didn’t necessarily think that [vendor] is better than what is being provided…In dollars and cents wise, is it funneling off billable encounters from us? And, if that’s the case, then why?"

(Clinic Provider, FG4)

Other concerns were related to workflow and logistics. One administrator was concerned about division of responsibility between clinic and vendor providers, and potential burden:

“These are gonna be our patients, when they turn up in crisis and can’t have immediate access to these telehealth providers. We’re gonna be the ones dealing with them, so why shouldn’t we be the ones doing all of their care?"

(Clinic Administrator, SI4)

Vendor personnel suggested that this model requires pre-referral communication between clinic and TM providers about what clinics should expect from the vendor and about the patients being referred. Vendor personnel and providers noted that communication problems hampered implementation of the model during the feasibility study due to different philosophies and prescribing practices from clinics. These issues led to lack of trust between primary care and vendor providers, and vendor providers also mentioned that limited communication between vendor and primary care providers could (and did) lead to some patients “splitting” among their providers, sometimes providing different information to each provider. As one provider shared:

"There has been—because of—for the lack of a better term, probably I’m gonna call it power struggle. There has been a power struggle on which treatment model needs to be utilized, and that has caused confusion to the patients. Because now the patients do not know where they are getting most of their treatment…."

(Clinic Staff, TM29)

While most clinic and TM providers felt that pre-referral and ongoing communication could resolve conflict, some believed that communication after referral isn’t necessary:

“You referred them out because I am the addiction medicine doctor, so I don’t get involved with what you’re prescribing over there for blood pressure. Why are you in my lane?”

(Prescribing Vendor Provider, TM27)

TM-MOUD vendor providers also felt that a pragmatic collaboration plan, strong relationships and ongoing transparent communication between vendor and clinics are needed to implement and sustain the model. This TM-MOUD provider’s comment reflects the common sentiment that a collaborative model in which the patient is connected to both the clinic and the TM vendor staff and providers could ultimately be of great benefit to patients:

“[Patients are in the clinic] and they’re among people that they’re familiar with, and all we’re doing is offering the education and the support and the prescription.”

(Prescribing Vendor Provider, TM23)

*Theme 10: There are technical and communication barriers to the TM-MOUD referral and coordination model*. Participants thought that technical and communication issues could impede TM-MOUD implementation. Some perceived that communication issues between the vendor, the clinic, and patients resulted in longer than expected patient wait-times (e.g., waiting to be contacted by the vendor, waiting in a virtual “waiting room”). In some cases, inability of the vendor to contact patients resulted in the patient not starting or quickly after starting treatment, dropping out of treatment with the TM vendor. This patient expressed frustration with the process:

"Well no one told me that there was a certain process of how to check in. So me just saying, ‘I’m ready to check in,’ assuming it will identify me; no, you actually have to identify yourself. You have to identify that you’ve done the check-in process online—which appointment you’re checking into. So there’s been some communication issues.

(Patient, PI1)

Of note, TM-MOUD vendor participants suggested that technological issues are the biggest barrier to the model and to TM-MOUD. Examples included patients having connectivity issues due to lack of adequate Wi-Fi, lack of access to technology, and low technological literacy. While clinic and TM-MOUD vendor personnel expressed concern about technical challenges, patients did not express this concern.

*Theme 11: Filling prescriptions in local pharmacies can be difficult for TM providers*. A final barrier to TM-MOUD more generally noted by several TM-MOUD prescribing providers was that it can be difficult to get local pharmacies to fill prescriptions from a TM-MOUD provider. TM-MOUD providers explained that some pharmacies are resistant to filling prescriptions for buprenorphine/naloxone specifically, which participants noted could be due to stigma toward people with OUD and toward buprenorphine treatment more generally, than to TM-MOUD specifically. In addition, TM-MOUD providers noted that some pharmacies won’t fill a prescription for a controlled substance from a provider they don’t know or for an out-of-state provider, as described by this TM-MOUD provider:

"I have challenges with the pharmacies a lot. They don’t like my multiple licenses. They’ll tell me all the time, ‘You have to be local.’ There’s challenges to where they think—you know, we’re not real doctors or we don’t have real licenses. That’s going away as we get more and more, uh—as time goes by, it goes away, and I take less offense to it."

(Prescribing Vendor Provider, TM28)

### 3.3. Acceptability ratings for TM-MOUD elements

**Fig. 2.**
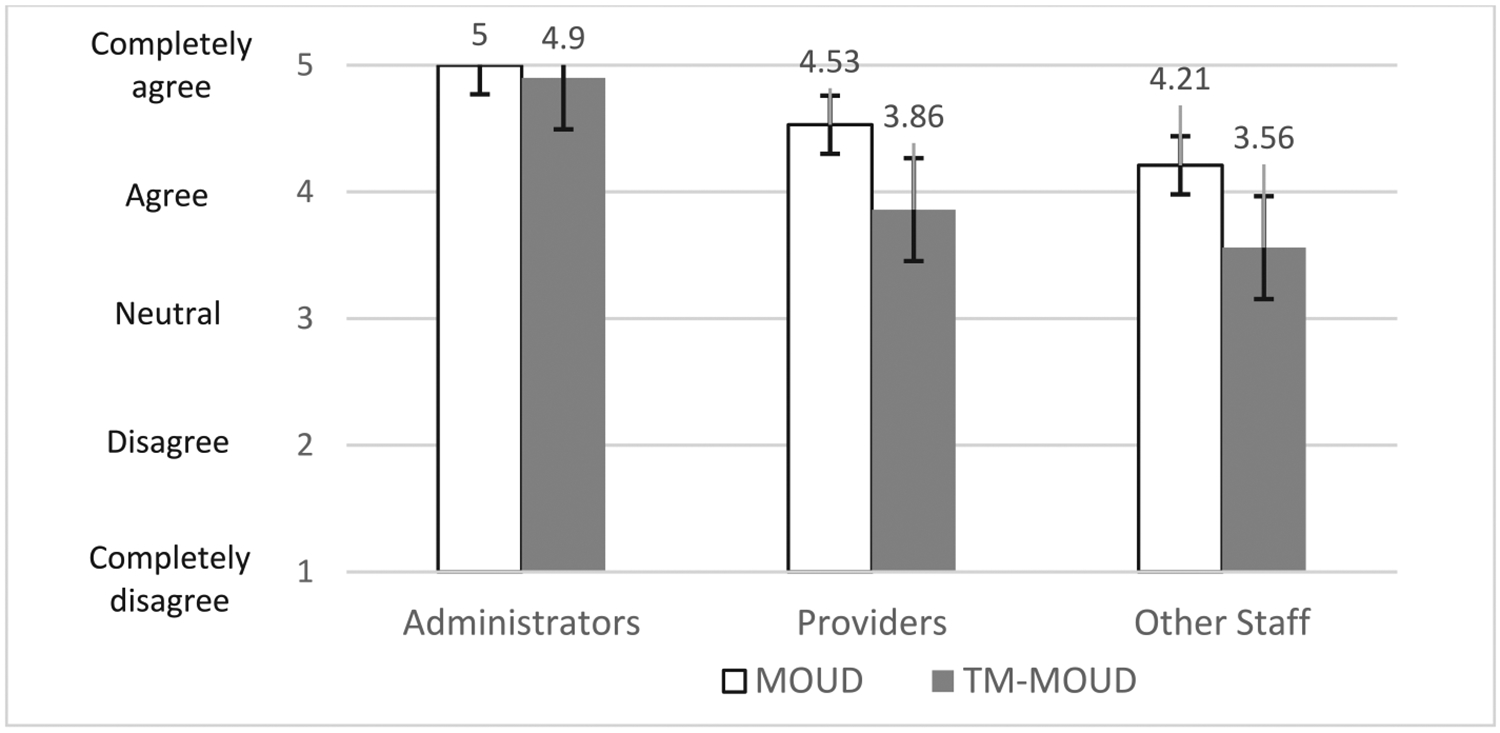
Acceptability ratings of MOUD and TM-MOUD: Clinic Administrators, Providers and Other Staff. Note. *N* (Administrators) = 5, *N* (Providers) = 24, *N* (Clinic Staff) = 33. Error bars indicate the 95 % confidence interval around each mean rating. OUD = opioid use disorder, MOUD = Medication for OUD, TM = telemedicine.

Fig. 2 summarizes *clinic personnel* perceptions of overall acceptability for MOUD generally (i.e., MOUD services provided at the clinic, including induction, medication management and recovery support) and TM-MOUD (i.e., MOUD services provided via live video or telephone). The figure shows the mean ratings of each element for providers, staff, and administrators. Mean scores for MOUD were highest among administrators (*M* = 5.00, *SD* = 0.0), followed by providers (*M* = 4.53, *SD* = 0.86), and then other staff (*M* = 4.21, *SD* = 0.70). Average ratings for TM-MOUD (generally, not specific to the study vendor) were highest among administrators (*M* = 4.90, *SD* = 0.31), followed by providers (*M* = 3.86, *SD* = 0.99), and then other staff (*M* = 3.56, *SD* = 1.00).

**Fig. 3.**
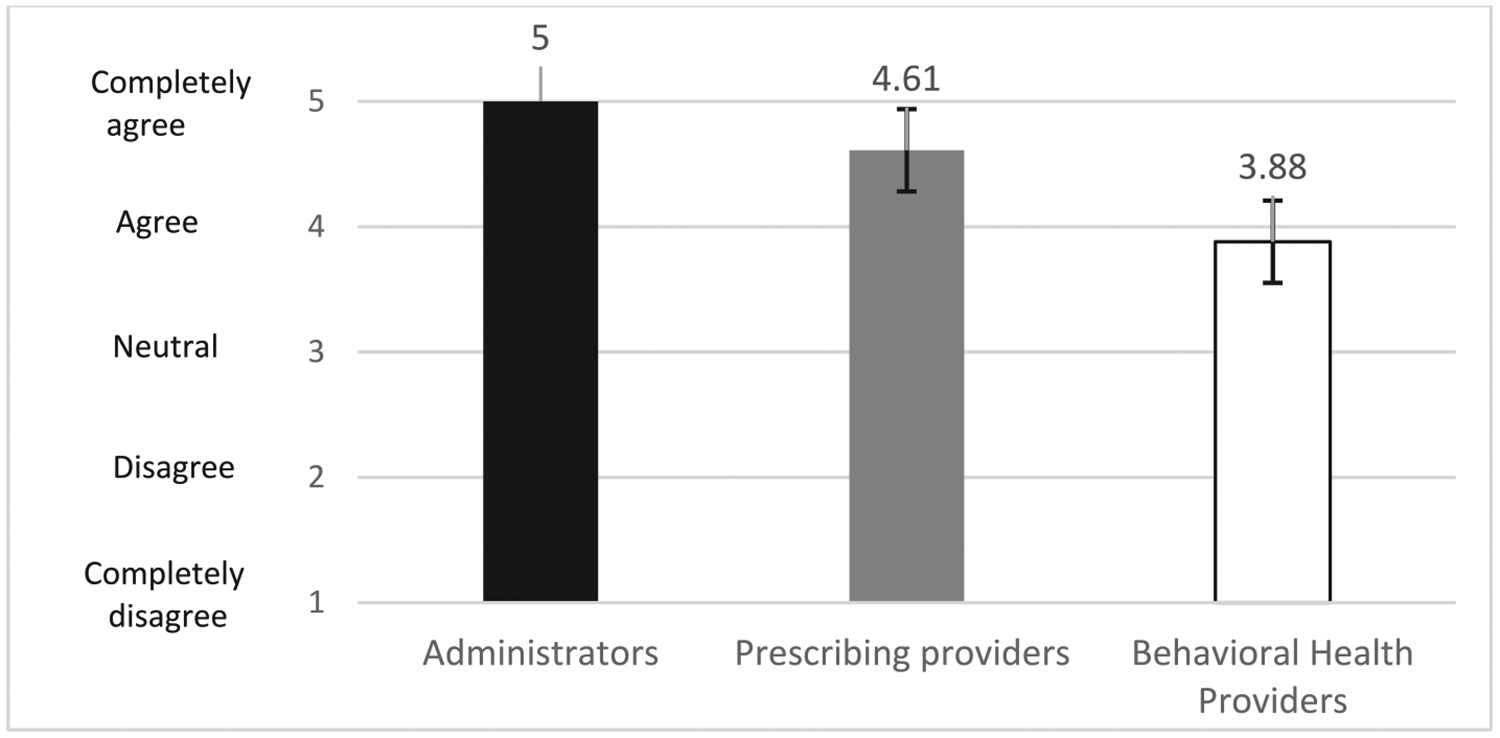
Acceptability ratings for the TM-MOUD referral and care coordination model: Vendor administrators, prescribing and behavioral health providers. Note. *N* (Administrator) = 4, N (prescribing provider) = 9, N (Behavioral Health) = 8. Error bars indicate the 95 % confidence interval around each mean. MOUD = Medication for opioid use disorder, TM = telemedicine.

Fig. 3 shows *acceptability ratings for the TM-MOUD referral and coordination model among TM vendor administrators and staff, prescribers, and behavioral health providers*. Ratings were highest among administrative staff and others (*M* = 5.0, *SD* = 0.0), followed by prescribers (*M* = 4.51, *SD* = 1), and then behavioral health providers (*M* = 3.88, *SD* = 1).

**Fig. 4.**
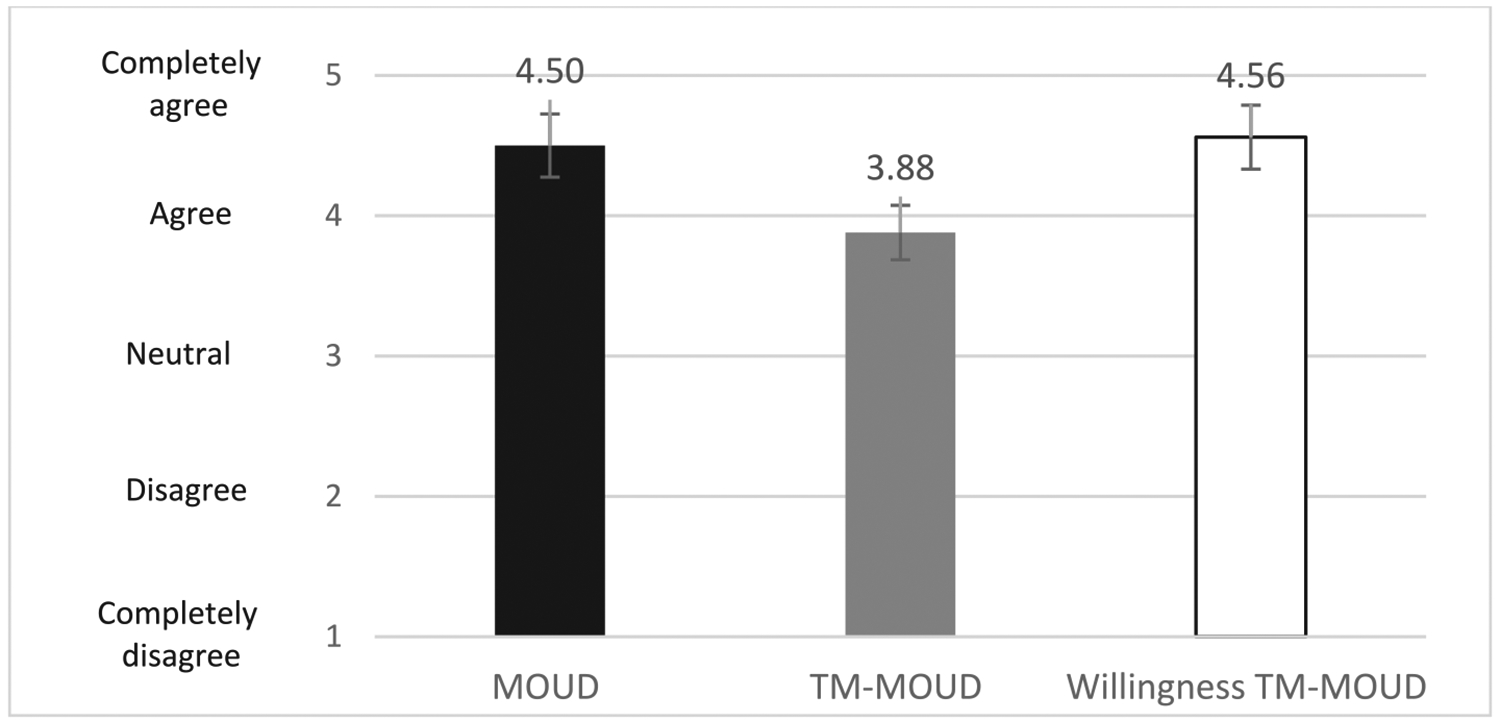
Patient acceptability ratings for MOUD and TM-MOUD and willingness to try TM-MOUD. Note. *N* = 16. Error bars indicate the 95 % confidence interval around each mean rating. OUD = opioid use disorder, MOUD = Medication for OUD, TM = telemedicine.

Fig. 4 summarizes *patient acceptability ratings* of MOUD, TM for MOUD, i.e., opinions about MOUD and TM-MOUD services generally, and patients’ willingness to try TM-MOUD themselves. Mean ratings were high for MOUD (*M* = 4.50, *SD* = 0.73) and for patients’ willingness to try TM-MOUD (*M* = 4.56, *SD* = 0.63); ratings about TM-MOUD were lower (*M* = 3.88, *SD* = 1.15).

## 4. Discussion

This study explored stakeholder experiences of MOUD implementation generally and of a TM-MOUD referral and coordination model to help expand MOUD treatment in rural primary care settings. Participants acknowledged the benefits of the TM-MOUD referral and coordination model to clinics and to patients with OUD, but also suggested that several key factors must be in place to facilitate successful implementation and sustainability of such a model. Themes from the interviews and focus groups suggest that clinic buy-in and preparation are essential to implementing the referral and coordination model with a TM-MOUD vendor, along with pre-referral and ongoing communication and collaboration between clinic and TM providers and with patients.

Although healthcare services conducted via an external TM vendor or health system (e.g., endocrinology, rheumatology, dermatology, psychiatry) is a common and growing practice in rural and low-resource settings (Uscher-Pines, Sousa, Palimaru, et al., 2020), our findings suggest that referral to an external TM-MOUD vendor from a primary care setting may bring unique complexities. Complexities echo those found in prior studies of MOUD implementation more generally, and may be due to the nature of OUD and its treatment, such as stigma toward people with OUD and toward MOUD (Storholm et al., 2017), and challenges engaging patients with substance use disorders in treatment (Krawczyk et al., 2021). Additionally, our study suggests that integrating a TM-MOUD referral and coordination model into busy primary care practices can take significant time and effort, as with any new practice (Grantham et al., 2017; Green et al., 2009). MOUD also may not be accepted by all providers in the primary care setting because rural healthcare services have important differences compared to urban or suburban settings, where many TM providers are located (van Dis, 2002) and where most TM service expansion has occurred during the ongoing COVID pandemic (Cantor et al., 2021; Chu et al., 2021). Overall, TM referral and care coordination for OUD may be qualitatively different from other TM specialty care models in unique ways given the complex nature of addiction treatment; ways that may require greater planning and coordination to address, with continued attention to barriers and facilitators to MOUD generally (which were prominent in the themes).

Even when well-planned with effective communication in place, participants in this study suggested that technological barriers and patients’ hesitation to engage in treatment more generally must be addressed to make TM-MOUD referral and coordination with primary care successful. Most stakeholders, but especially those affiliated with the TM-MOUD vendor, noted that technological barriers are the single most substantial barrier to TM-MOUD, with lack of Wi-Fi access, low technological capacity among patients, and lack of adequate devices among them. Lack of adequate Wi-Fi connectivity is a structural issue, and might be improved with the federal government’s recent allocation of $65 billion to improve broadband infrastructure in rural areas (U.S. Senate - Commerce, 2021). Of note, patients in this study did not express technology as a barrier, but this might be because the patients who agreed to be interviewed were already well-connected to providers through telephone and email, and some through video TM. Patients’ variable readiness for treatment and related challenges around MOUD adherence and appointment attendance are common experiences for people with substance use disorders (Krawczyk et al., 2021). Some TM-MOUD providers believed that TM “no-show” rates are similar to those experienced in brick-and-mortar settings and feel that TM alone cannot solve engagement challenges. Notably, clinic patients currently taking MOUD and some TM-MOUD providers in this study felt that TM-MOUD would be best for patients already induced and stabilized on MOUD, citing accountability and compliance issues as potentially more problematic with TM-MOUD than in a brick-and-mortar setting. Further research is needed to determine what types of patients can succeed best through TM-MOUD. For some patients it may be the only treatment available, in which case some treatment may be better than no treatment at all, while for others the challenges of TM-MOUD may be greater than those of receiving in-person care, where it is available. Whether in-person or via TM, continued development of flexible service models for OUD and other substance use disorders that balance engagement of patients with feasibility for providers is needed.

In addition to TM-MOUD, clinic participants also discussed their perspectives about elements of the continuum of OUD care within the clinic that are needed for successful implementation both of clinic-based MOUD and referral to TM-MOUD. Clinic personnel and patients felt screening was worthwhile and necessary for identifying patients with OUD and transitioning them to treatment. However, implementing new screening practices for patients’ opioid use was problematic during the feasibility study. Barriers were similar to those identified in prior studies, including workflow implications, the need to adapt practices to find the best fit for the clinic, and the need for time to implement and test alternatives (McNeely et al., 2018; Rahm et al., 2015). Additionally, concerns about patients not answering screening questions truthfully reflect barriers also noted by others implementing OUD screening in primary care (McNeely et al., 2018). Normalizing screening and conducting it annually, increasing privacy during screening, and reducing stigma through provider training and education may help mitigate these issues (McNeely et al., 2018). Regular screening for unhealthy drug use including OUD in primary care settings is important and recently was recommended by the U.S. Preventive Services Task Force, with experts noting that any discomforts experienced during screening are outweighed by the benefits, which include referring patients to appropriate evidence-based care (McNeely, 2020; Patnode et al., 2020). Screening can be optimized by offering screening at any visit using self-administered screening tools (McNeely et al., 2021). Diagnosing OUD, the next step in successfully managing patients with OUD, was not discussed directly; however, correctly entering OUD in the EHR was identified as problematic due to inconsistencies with how OUD diagnoses are coded and documented. Indeed, other studies have found that DSM-5 diagnoses within medical records may be incorrect; one study suggests that it may be possible to examine other data within the medical record (e.g., past prescriptions for opioids) to help identify people with OUD and provide accurate diagnoses (Palumbo et al., 2020).

Our findings, which are based primarily on qualitative data, suggest acceptability and feasibility among staff and patients of MOUD generally and TM-MOUD; quantitative ratings highlight nuances identified in the qualitative data. For example, the lower quantitative ratings of MOUD generally and TM-MOUD among clinic staff (e.g., medical assistants, registered nurses at the clinic) other than administrators and prescribers may reflect that these clinic personnel were tasked with much of the work around managing clinic-based MOUD scheduling generally and coordinating with the TM-MOUD vendor during the study. As such, these staff were more frequently confronted by immediate patient challenges, while administrators and providers typically were less involved in those procedures. Similarly, we speculate that lower ratings of TM-MOUD referral and coordination by TM vendor behavioral health staff may reflect more limited acceptability among providers who generally hold responsibility for engaging patients in treatment activities, or that patients may have wanted TM more for medication than for behavioral health treatment, although behavioral health staff did not report on this directly during our interviews. For the patient ratings, lower ratings for TM-MOUD services generally, compared with their own willingness to try TM-MOUD, may be due to their views that TM-MOUD is better for patients who are “more stable” on MOUD. Patients who participated in interviews mostly were already taking MOUD, some for many years, and may have viewed themselves as more stable and better suited for TM than a newer OUD patient. Providers’ descriptions of “stable” patients were inconsistent, so it is important to involve patients in decision-making rather than making assumptions about their likely success with MOUD and/or TM. These nuances emphasize the need for understanding differences in perceptions of these practices among different types of staff and among patients.

Our study has several limitations. First, the timeline for this feasibility study coincided with the start of the COVID-19 pandemic in 2020, providing important context for the conditions within which the research team, partner clinics, and TM-MOUD vendor were operating, and possibly impacting perspectives about TM-MOUD. During this time, partner clinics were making major adjustments to clinic operations and MOUD treatment to minimize COVID-19 risks – including providing primary care and MOUD treatment via TM – and the research team modified implementation and data collection activities from in-person to virtual formats. By the time we collected data, most participants also had some level of direct experience with TM, whereas many had no experience with TM prior to 2020. Second, due to pandemic-related delays and lack of ability to collect data in person, we were not able to collect pre- and post-study data; instead, we collected data throughout the study period, which resulted in some stakeholders having more experience with the TM-MOUD referral and coordination model than others. Nevertheless, the pandemic also raised awareness of TM among primary care staff and created a greater and more immediate need for it.

Next, participants volunteered to participate in qualitative data collection and surveys, so self-selection bias may exist in reported experiences and support for the TM-MOUD model. Furthermore, the study worked with a single TM-MOUD vendor, and both the vendor and health systems primarily focused on buprenorphine for MOUD treatment. Thus, perceptions of this model may not generalize to other TM vendors or health systems that provide MOUD and may not have captured important barriers and facilitators of using TM to support other treatments like naltrexone or methadone. Also, clinic providers and patients were recruited from three states, so findings may not be generalizable to other geographic regions, and subsamples of staff types were small—perhaps too small to reach theme saturation. Further, it is possible we did not uncover all topics that could potentially impact implementation of TM-MOUD, but we did cover those most salient for participants of this study.

Finally, the provider and patient samples lacked racial and ethnic diversity, perhaps reflecting lack of diversity in the communities where the study was taking place. Future research in rural settings must aim for a more diverse sample. Nevertheless, we had ample data to reach saturation on themes and did obtain a range of perspectives that overlapped among stakeholder groups. Future research should examine different models of working with vendors outside of primary care (such as direct referral without coordination), identifying which patients are best served by TM-MOUD, and determining how TM-MOUD compares with in-person treatment for patient experience and outcomes.

## 5. Conclusions

Despite some differences in opinions about and barriers to implementing a TM-MOUD referral and coordination model, most stakeholders ultimately agreed that the model is beneficial and could be the best option for certain patients, particularly for those with transportation issues and in communities that lack resources to provide needed treatment, and for those who are tech-savvy with few technological barriers. As with implementation of any new practice into a healthcare setting, implementing this TM-MOUD referral and coordination model requires buy-in not only from primary care clinic staff, providers, leadership, and patients, but also from TM-MOUD vendor staff and providers. Clinic staff and providers need to be knowledgeable in advance about MOUD and the TM-MOUD workflow and services offered; how, when and how much clinical information will be shared by the TM-MOUD provider with the clinic; what types of patients are most appropriate for referral; and about agreed upon protocols and professional boundaries regarding prescribing practices between clinic and TM-MOUD prescribers and about dispute management, in the event of disagreement. Similarly, TM-MOUD vendor staff and providers need to know what clinical information to share with clinics and how and when to share. Patients should be informed about TM-MOUD vendor requirements and expectations for program participation and about potential wait-times. While a TM-MOUD referral and coordination model is not the only way to improve access to OUD treatment for people in rural communities, offering such an option through primary care or outside of primary care may be one way to improve access to and retention on MOUD for patients as part of broader efforts to address rural health disparities and prevent opioid-related deaths.

## Figures and Tables

**Table 1:** Demographics.

Characteristic	All Respondents		Clinic Providers		Clinic Staff		Clinic Administrators		Clinic Patients		TM Staff & Providers	
	(*N* = 99)		(*N* = 24)		(*N* = 33)		(*N* = 5)		(*N* = 16)		(*N* = 21)	
Age M (SD)	45.42	(12.51)	45.9	(11.73)	42.8	(12.57)	52.6	(8.71)	39.86	(11.54)	51.56	(12.52)
	N	%	N	%	N	%	N	%	N	%	N	%
Gender^a^												
Male	25	25 %	9	38 %	1	3 %	2	40 %	7	44 %	6	29 %
Female	73	74 %	15	63 %	32	97 %	3	60 %	9	56 %	14	67 %
Unknown/Missing	1	1 %	0	0 %	0	0 %	0	0 %	0	0 %	1	5 %
Ethnicity												
Not Hispanic or Latino	75	76 %	23	96 %	31	94 %	5	100 %	16	100 %	0	0 %
Hispanic or Latino	2	2 %	1	4 %	1	3 %	0	0 %	0	0 %	0	0 %
Unknown/Missing	22	22 %	0	0 %	1	3 %	0	0 %	0	0 %	21	100 %
Race												
White (no other race selected)	81	82 %	22	92 %	29	88 %	5	100 %	13	81 %	12	57 %
Non-White or >than one race^b^	14	14 %	0	0 %	3	9 %	0	0 %	3	19 %	8	38 %
Unknown/Missing	4	4 %	2	8 %	1	3 %	0	0 %	0	0 %	1	5 %
Education												
High school/GED or less	7	7 %	0	0 %	1	3 %	0	0 %	6	38 %	0	0 %
Some college, no degree	23	23 %	0	0 %	14	42 %	0	0 %	8	50 %	1	5 %
Associate degree	17	17 %	0	0 %	13	39 %	2	40 %	2	13 %	0	0 %
Bachelor’s degree	9	9 %	2	8 %	3	9 %	0	0 %	0	0 %	4	19 %
Graduate or medical degree	43	43 %	22	92 %	2	6 %	3	60 %	0	0 %	16	76 %
Region												
Northeast	40	40 %	12	50 %	16	48 %	3	60 %	9	56 %	N/A	N/A
Pacific Northwest	38	38 %	12	50 %	17	52 %	2	40 %	7	44 %	N/A	N/A
Unknown/Missing	21	21 %	0	0 %	0	0 %	0	0 %	0	0 %	N/A	N/A

Note. Demographic data collected from the 99 participants who completed the survey.

a Participants could respond “male,” “female,” or “trans/nonbinary.”

b Due to the low number of responses in race categories other than White (only), all “non-white” races and multiple races were combined for reporting purposes.
